# Cytosolic phospholipase A_2_: a member of the signalling pathway of a new G protein α subunit in *Sporothrix schenckii*

**DOI:** 10.1186/1471-2180-9-100

**Published:** 2009-05-19

**Authors:** Shirley Valentín-Berríos, Waleska González-Velázquez, Lizaida Pérez-Sánchez, Ricardo González-Méndez, Nuri Rodríguez-del Valle

**Affiliations:** 1 Department of Microbiology, San Juan Bautista School of Medicine, Box 4968, Caguas, PR 00726-4968, USA; 2 Department of Microbiology and Medical Zoology, Medical Sciences Campus, University of Puerto Rico, PO Box 365067, San Juan, PR 00936-5067, USA; 3 Department of Radiological Sciences, Medical Sciences Campus, University of Puerto Rico, PO Box 365067, San Juan, PR 00936-5067, USA

## Abstract

**Background:**

*Sporothrix schenckii *is a pathogenic dimorphic fungus, the etiological agent of sporotrichosis, a lymphocutaneous disease that can remain localized or can disseminate, involving joints, lungs, and the central nervous system. Pathogenic fungi use signal transduction pathways to rapidly adapt to changing environmental conditions and *S. schenckii *is no exception. *S. schenckii *yeast cells, either proliferate (yeast cell cycle) or engage in a developmental program that includes proliferation accompanied by morphogenesis (yeast to mycelium transition) depending on the environmental conditions. The principal intracellular receptors of environmental signals are the heterotrimeric G proteins, suggesting their involvement in fungal dimorphism and pathogenicity. Identifying these G proteins in fungi and their involvement in protein-protein interactions will help determine their role in signal transduction pathways.

**Results:**

In this work we describe a new G protein α subunit gene in *S. schenckii*, *ssg-2*. The cDNA sequence of *ssg-2 *revealed a predicted open reading frame of 1,065 nucleotides encoding a 355 amino acids protein with a molecular weight of 40.9 kDa. When used as bait in a yeast two-hybrid assay, a cytoplasmic phospholipase A_2 _catalytic subunit was identified as interacting with SSG-2. The *sspla*_*2 *_gene, revealed an open reading frame of 2538 bp and encoded an 846 amino acid protein with a calculated molecular weight of 92.62 kDa. The principal features that characterize cPLA_2 _were identified in this enzyme such as a phospholipase catalytic domain and the characteristic invariable arginine and serine residues. A role for SSPLA_2 _in the control of dimorphism in *S. schenckii *is suggested by observing the effects of inhibitors of the enzyme on the yeast cell cycle and the yeast to mycelium transition in this fungus. Phospholipase A_2 _inhibitors such as AACOCF3 (an analogue of archidonic acid) and isotetrandrine (an inhibitor of G protein PLA_2 _interactions) were found to inhibit budding by yeasts induced to re-enter the yeast cell cycle and to stimulate the yeast to mycelium transition showing that this enzyme is necessary for the yeast cell cycle.

**Conclusion:**

A new G protein α subunit gene was characterized in *S. schenckii *and protein-protein interactions studies revealed this G protein alpha subunit interacts with a cPLA_2 _homologue. The PLA_2 _homologue reported here is the first phospholipase identified in *S. schenckii *and the first time a PLA_2 _homologue is identified as interacting with a G protein α subunit in a pathogenic dimorphic fungus, establishing a relationship between these G proteins and the pathogenic potential of fungi. This cPLA_2 _homologue is known to play a role in signal transduction and fungal pathogenesis. Using cPLA_2 _inhibitors, this enzyme was found to affect dimorphism in *S. schenckii *and was found to be necessary for the development of the yeast or pathogenic form of the fungus.

## Background

*Sporothrix schenckii *is a dimorphic fungus that produces lymphocutaneous lesions in humans and animals. It is the etiologic agent of sporotrichosis, a subcutaneous lymphatic mycosis with a worldwide distribution [1]. In its saprophytic form it develops hyaline, regularly septated hyphae and pyriform conidia which can be found single or in groups in a characteristic daisy-like arrangement. The yeast or parasitic form shows ovoid cells with single or multiple budding.

In *S. schenckii*, dimorphism is both a proliferative and morphogenetic process. We have reported that in response to different environmental stimuli, *S. schenckii *unbudded synchronized yeast cells, either proliferate (yeast cell cycle) or engage in a developmental program that includes proliferation accompanied by morphogenesis (yeast to mycelium transition). Dimorphism in *S. schenckii*, depends on transmembrane signalling pathways that respond to cell density [2,3], external pH [2,3], cyclic nucleotides [4] and extracellular calcium concentration [5].

Dimorphism is an adaptation response to changing environmental conditions. The morphology displayed by dimorphic fungi is probably the result of the stimulation of membrane receptors by extracellular ligands. Heterotrimeric (αβγ) guanine nucleotide binding proteins have been associated with membrane receptors and with morphogenetic transition signalling in many eukaryotes, and play a crucial role in fungal morphogenesis as well [6]. They constitute a family of GTP hydrolases involved in signal transduction pathways. These proteins are coupled to membrane receptors (GPCR) that recognize different extracellular signals. The α subunits of the heterotrimeric G proteins bind GTP. The interaction of a ligand with the GPRC initiates the exchange of bound GDP for GTP in the Gα subunit resulting in the dissociation of the heterotrimer into α-GTP and βγ subunits. The dissociated α-GTP subunit and the βγ dimer, relay signals to different targets resulting in changes in cytoplasmic ionic composition or in second messenger levels (e.g., cAMP) that ultimately lead to a cellular response [7-10].

Genes encoding proteins that are similar to the Gα class of the heterotrimeric G proteins have been described in filamentous fungi such as *Aspergillus nidulans *[11] and *Neurospora crassa *[12-14], as well as in fungal plant pathogens like *Cryphonectria parasitica *[15,16], *Ustilago maydis *[17] and *Magnaporthe grisea *[18], among others. In *S. schenckii*, a 41 kDa Gα subunit homologous to the Gαi subunit and sensitive to inhibition by pertussis toxin was described previously by us [19]. This was the first Gαi subunit described in a pathogenic dimorphic fungus.

In higher eukaryotes, members of the Gα class are known to regulate adenylate cyclase [20], cGMP phosphodiesterase [21], phosphoinositide-3-kinase [22], calcium and potassium channels [22-24], and the activity of phospholipases [9,25-28]. In fungi, Gα subunits have been shown to regulate adenylate cyclase, morphogenesis and pathogenicity [6,14,29,30]. Most of the studies related to determining the role of the heterotrimeric G protein subunits in fungi involved the observation of the morphological effects produced in the fungus when these genes are deleted [6,12,14,18]. Nevertheless, the full scope of the processes that Gα subunits regulate in fungi is still not known and interactions between these subunits and cellular proteins have seldom been reported in pathogenic fungi.

A large number of G protein coupled receptors have been observed to induce activation of phospholipase A_2 _in higher eukaryotic systems [31]. The PLA_2 _superfamily can be classified according to cellular location or biological properties [32]. The phospholipase A superfamily includes the calcium dependent-secretory PLA_2 _(sPLA_2_), the calcium independent-intracellular PLA_2 _(iPLA_2_) and the cytosolic PLA_2 _(cPLA_2_). They differ in terms of calcium requirements, substrate specificity, molecular weight and lipid modification. The sPLA_2 _is usually a 13 to 15 kDa protein while the cPLA_2 _is a 85 kDa protein in human macrophages. The cPLA_2 _possesses characteristics that suggest that it is associated to receptor-activated signal transduction cascades [33]. This PLA_2 _is known to translocate to the membrane in response to an increase in intracellular calcium concentration [34]. Cytosolic PLA_2 _hydrolyses the *sn*-2 position of phospholipids, resulting in the release of lysophospholipids and free fatty acids. The most commonly released fatty acid is arachidonic acid, which in turn is converted to eicosanoids that regulate multiple processes including calcium channels, mitogenic signals and most important, the inflammatory response of macrophages [31,32,35,36].

The present study was undertaken to identify the presence of and characterize additional Gα subunits in *S. schenckii*, to identify any important interacting partners of the new Gα subunit, and finally to determine the involvement if any of the interacting protein, in this case cPLA_2_, in the control of dimorphism in this fungus. Here we give details of the identification and sequencing of the *ssg-2 *gene, including gene organization, the presence and position of introns, derived amino acid sequence and conserved polypeptide-encoded domains. Using SSG-2 as bait, we identified a cPLA_2 _homologue interacting with this G protein α subunit. We give the genomic sequence of this gene and the complete derived amino acid sequence. We also report the effects on the yeast to mycelium transition and the yeast cell cycle of phopholipase effectors in *S. schenckii*.

This work constitutes the first report of the presence of multiple G protein α subunits in *S. schenckii*, the presence of a cPLA_2 _homologue interacting with this G protein α subunit, and the involvement of cPLA_2 _in the control of dimorphism in *S. schenckii*. In addition to being a very important determinant of pathogenicity in fungi and other organisms, cPLA_2 _is shown to have a direct effect in the control of dimorphism in this fungus. This information will ultimately help us construct the signal transduction pathway leading from the G proteins onward and the role of G proteins and its interacting partners in fungal pathogenesis.

## Results

### Identification of the *ssg-2 *gene

**Figure 1:**
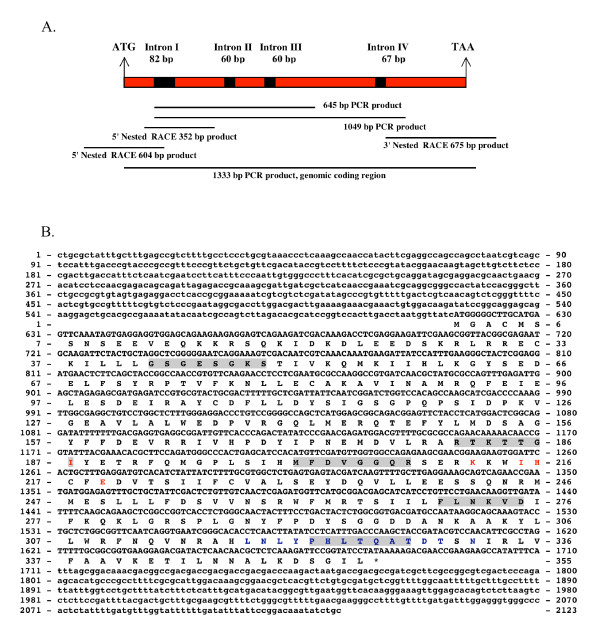
**cDNA and derived amino acid sequences of the *S. schenckii ssg-2 *gene**. Figure 1A shows the sequencing strategy used for *ssg-2*. The size and location in the gene of the various fragments obtained from PCR and RACE are shown. The black boxes indicate the size and relative position of the introns. Figure 1B shows the cDNA and derived amino acid sequence of the *ssg-2 *gene. Non-coding regions are given in lower case letters, coding regions and amino acids are given in upper case letters. The sequences that make up the GTPase domain are shaded in gray, the five residues that identify the adenylate cyclase interaction site are given in red and the putative receptor binding site is shown in blue.

Most fungal Gα subunit genes vary only slightly in size within the region encoding the GESGKST and KWIHCF motifs where primers for PCR are usually made because of the conserved nature of these regions. In the region comprised between these primers size variations are usually due to the presence of introns of slightly different sizes. Two PCR products were obtained when using fungal DNA as template and the GESGKST/KWIHCF primer pair one belonging to *ssg-1 *and the other to *ssg-2 *of approximately 620 and 645 bp, respectively. The *ssg-2 *PCR product (645 bp) established the presence of a new gene encoding another Gα subunit in *S. schenckii*. Figure 1A shows the sequencing strategy used for the identification of this new G protein α subunit gene. Once the coding sequence was completed, it was confirmed using yeast cDNA as template and the MGACMS/KDSGIL primer pair. A 1,065 bp ORF was obtained, containing the coding region of the *ssg-2 *cDNA as shown in Figure 1B. Using the same primer pair and genomic DNA as template a 1,333 bp PCR product was obtained. Sequencing of this PCR product confirmed the sequences obtained previously and showed the presence and position of 4 introns. These introns had the consensus GT/AG junction splice site and interrupted the respective codons after the second nucleotide. The first intron interrupted the codon for G42 and consisted of 82 bp, the second intron interrupted the codon for Y157 and consisted of 60 bp, the third intron interrupted the codon for H200 and consisted of 60 bp, the fourth intron starts interrupted the codon H323 and consisted of 67 bp. With the exception of the regions where introns were present in the genomic sequence of the *ssg-2 *gene, the cDNA sequence and genomic sequence were identical. The overlapping of these two sequences confirmed the presence of the introns in the genomic sequence. The cDNA and genomic sequence of *ssg-2 *have GenBank accession numbers AF454862 and AY078408, respectively.

### Bioinformatic characterization of SSG-2

The derived amino acid sequence (GenBank accession number AAL57853) revealed a Gα subunit of 355 amino acids as shown in Figure 1B. The calculated molecular weight of the *ssg-2 *gene product was 40.90 kDa. Blocks analysis of the amino acid sequence of SSG-2 revealed a G-protein alpha subunit signature from amino acids 37 to 276 with an E value of 5.2e^-67 ^and a fungal G-protein alpha subunit signature from amino acids 61 to 341 with an E value of 3.3e^-28 ^[37]. SSG-2 has the motifs encoding the GTPase domain [38] with the corresponding consensus sequences involved in GTP binding shaded in gray in Figure 1B. The phosphate binding loop which includes the sequence GXGXXGKS is found in SSG-2 as GSGESGKS. The magnesium binding residues with the consensus sequence DXXG is present as DVGG in SSG-2, while the guanine ring binding sites are those with the consensus sequence NKXD is present as NKVD. The TXAT consensus sequence is present as TQAT in SSG-2. Another region involved in phosphate binding includes the consensus sequence RXXT that in SSG-2 is present as RTKT. In addition to these conserved domains, the protein derived from the *ssg-2 *cDNA sequence has the N-terminal glycine that is myristoylated in Gα subtypes and is needed for membrane association. The 5 residues that identify the adenylate cyclase interaction site according to BLAST analysis [39] are in red in Figure 1, these include I187, K212, I215, H216, and E 219. The putative receptor binding site includes amino acids L318 to R334 and is shown in blue letters in Figure 1[39].

**Figure 2:**
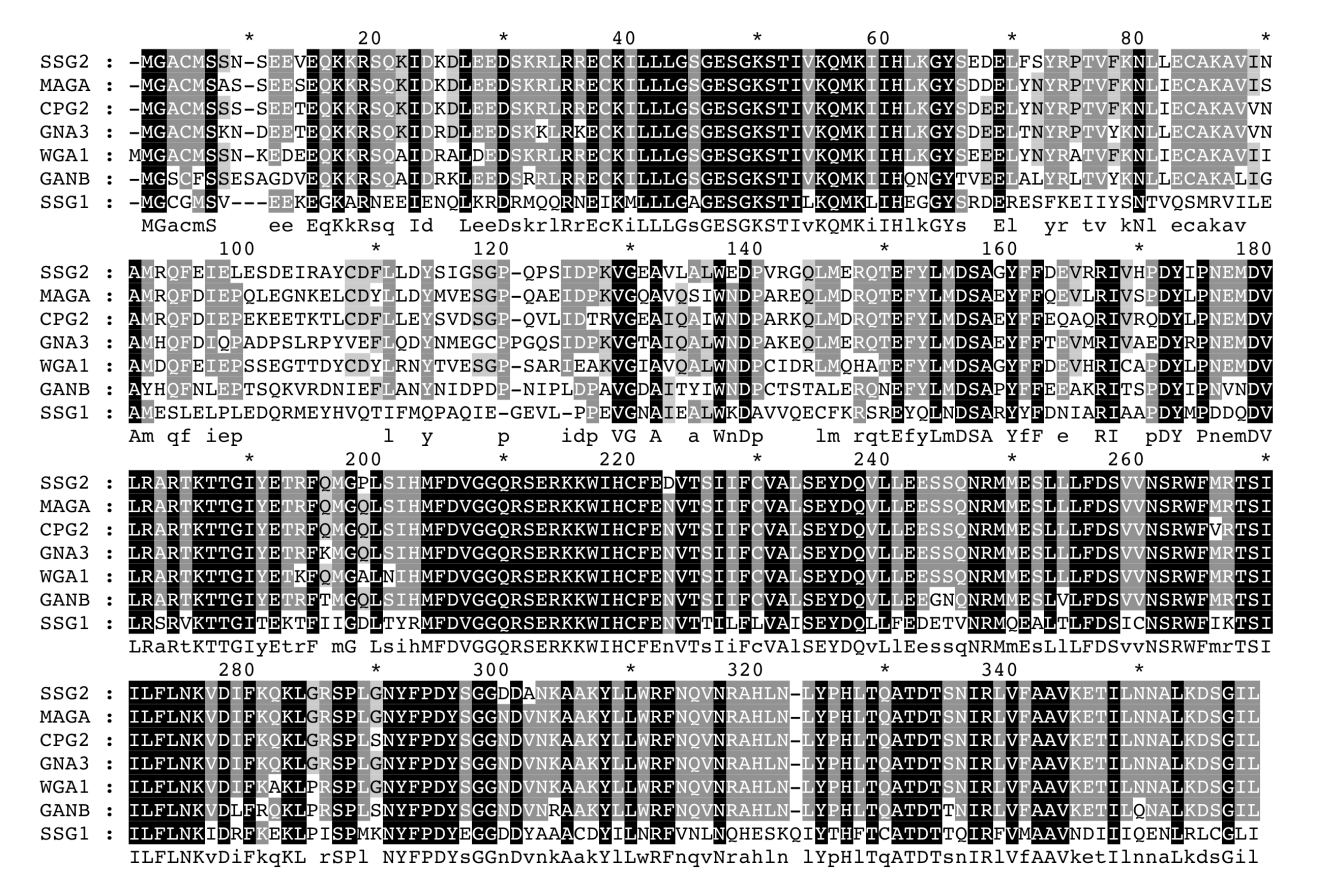
**Amino acid sequence alignments of SSG-2 with other Gα subunit homologues**. The predicted amino acid sequence of *S. schenckii *SSG-2 and SSG-1, *C. parasitica *CPG2, *N. crassa *GNA3, *R. necatrix *WGA1, *E. nidulans *GANB, and *M grisea *MAGA were aligned as described in Methods. In the alignment, black shading with white letters indicates 100% identity, gray shading with white letters indicates 75–99% identity, gray shading with black letters indicates 50–74% identity.

The derived amino acid sequence alignment of SSG-2 to that of the several fungal homologues is shown in Figure 2. This figure shows more than 85% identity to MAGA of *M. grisea *[18], CPG-2 of *C. parasitica *[16] and GNA-3 of *N. crassa *[14]. Table 1 summarizes the percent identity of SSG-2 to some members of the fungal Gα homologues and SSG-1.

**Table 1:** Comparison of G protein alpha subunit homologues to SSG-2 of *S. schenckii*

UniProt AC	Name	Length	Organism Name	Overlap	%iden	E-value	Score
Q8TF91	SSG2	355	*Sporothrix schenckii*	355	100	0	729
O13314	MAGA	356	*Magnaporthe grisea*	355	88	0	642
Q00581	CPG2	355	*Cryphonectria parasitica*	355	87	0	640
Q9HFW7	GNA3	356	*Neurospora crassa*	356	85	e-177	623
Q9HFA3	WGA1	356	*Rosellinia necatrix*	355	84	e-175	619
Q9UVK8	GANB	356	*Emericella nidulans*	356	77	e-160	567
O74259	SSG1	353	*Sporothrix schenckii*	353	50	2e-93	346

SSG-1 is included as reference. Analysis was carried out using iProtClass database and the BLAST algorithm. Overlap refers to the number of residues used to determine SSG-2% identity when doing pairwise comparisons.

### Yeast two-hybrid screening

Two independent yeast two-hybrid screenings, using different *S. schenckii *yeast cells cDNA libraries were done with the complete coding sequence of SSG-2 as bait. In both screenings, 3 blue colonies growing in quadruple drop out (QDO) medium (SD/-Ade/-His/-Leu/-Trp/X-α-gal) were identified as containing the same PLA_2 _homologue insert. The expression of the Ade^+^, His^+ ^phenotypes and α galactosidase activity are considered by the manufacturer as corroborative of true interactions. The inserts from all three colonies were found to contain the carboxy-terminal residues of a protein homologous to PLA_2_'s from *A. nidulans*. Our results indicated that the last 162 amino acids of the *S. schenckii *cPLA_2 _homologue interacted with SSG-2.

### Co-immunoprecipitation (Co-IP)

**Figure 3:**
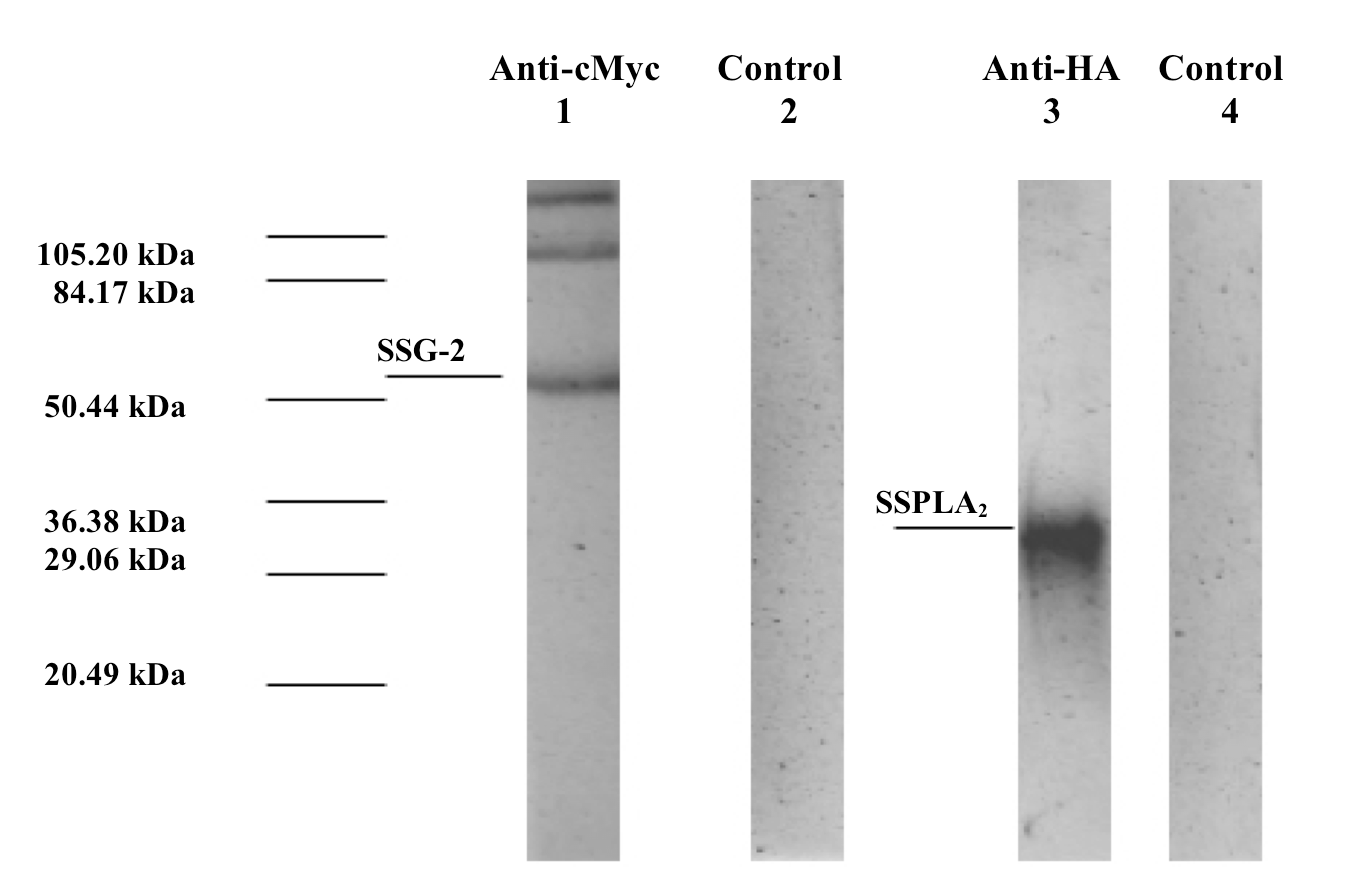
**Western Blots results from SSG-2/SSPLA_2 _co-immunoprecipitation**. Whole cell free extracts of *S. cerevisiae *cells containing PGBKT7 and PGADT7 plasmids with the complete SSG-2 coding region fused to the GAL4 activation domain and cMyc, and the initial SSPLA_2 _coding fragment identified in the yeast two-hybrid assay fused to the GAL4 DNA binding domain and HA, respectively, were co-immunoprecipitated as described in Methods. The co-precipitated proteins were separated using 10% SDS polyacrylamide electrophoresis and transferred to nitrocellulose. The nitrocellulose strips were probed with anti-cMyc antibodies (Lane 1) and anti HA antibodies (Lane 3). Lanes 2 and 4 are negative controls where no primary antibody was added. The antigen-antibody reactions were detected using the Immun-Star™ AP chemiluminescent protein detection system. Pre-stained molecular weight markers were included in outside lanes of the gel and also transferred to nitrocellulose, the position of the molecular weight markers is indicated in the figure.

The SSG-2-SSPLA_2 _interaction was corroborated by co-immunoprecipitation. Figure 3 shows the confirmation of the interaction observed in the yeast two-hybrid assay between SSG-2 and SSPLA_2 _by co-immunoprecipitation and Western blot analysis. Lane 1 shows the band obtained using anti-cMyc antibody that recognizes SSG-2. This band is of the expected size (62 kDa) considering that SSG-2 was expressed fused to the GAL-4 binding domain. The two high molecular weight bands present belong to the anti-cMyc antibodies used for precipitation. Lane 2 shows the results obtained in the Western blot when the primary anti-cMyc antibody was not added (negative control). Lane 3 shows the band obtained using anti-HA antibody that recognizes the original SSPLA_2 _fragment isolated from the yeast two-hybrid clone. This band is of the expected size (35.9 kDa) considering that only the last 162 amino acids of the protein were present and that this fragment was fused to the GAL-4 activation domain. Lane 4 shows the results obtained in the Western blot when the primary anti-HA antibody was not added (negative control).

### Sequencing of the *sspla*_*2 *_gene

**Figure 4:**
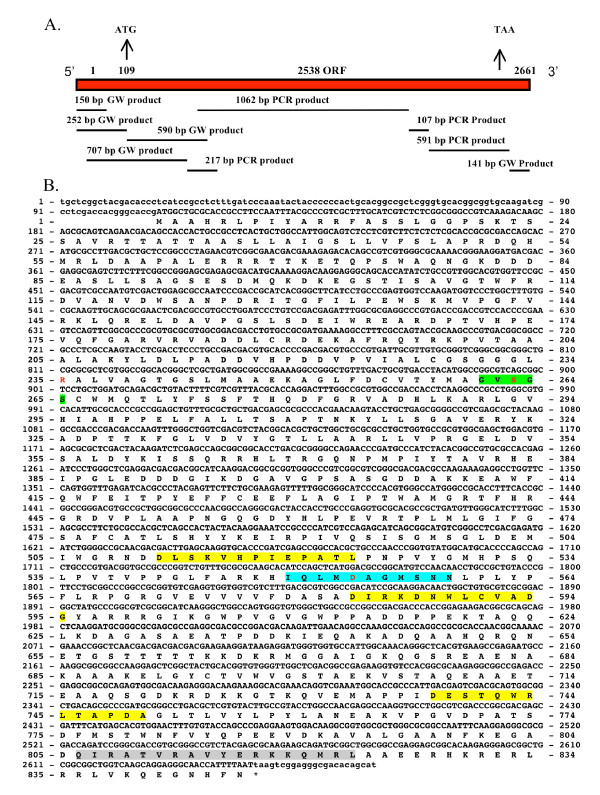
**Genomic and derived amino acid sequences of the *S. schenckii sspla*_*2 *_gene**. Figure 4A shows the sequencing strategy used for sequencing the *sspla*_*2 *_gene. The size and location in the gene of the various fragments obtained from PCR and RACE are shown. Figure 4B shows the genomic and derived amino acid sequence of the *sspla*_*2 *_gene. Non-coding regions are given in lower case letters, coding regions and amino acids are given in upper case letters. The invariant amino acids required for phospholipase activity are shown in red. The potential EF hands are shaded in yellow and the putative calmodulin binding domain is shaded in gray. The cPLA_2 _signature motif is shaded in green and the serine proteases, subtilase family, aspartic acid active site motif is shaded in blue green.

Figure 4A shows the sequencing strategy used for the *sspla*_*2 *_gene. The DNA sequence of *sspla*_*2 *_gene was completed using genome walking and PCR. Figure 4B shows the genomic and derived amino acid sequence of the *sspla*_*2 *_homologue. The genomic sequence has 2648 bp with an open reading frame of 2538 bp encoding an 846 amino acid protein with a predicted molecular weight of 92.6 kDa. The GenBank numbers for the genomic and derived amino acid sequence are FJ357242.1 and ACJ04517.1, respectively.

### Bioinformatic characterization of SSPLA_2_

The PANTHER Classification System identified this protein as a member of the cytosolic phospholipase A2 family (PTHR10728) (residues 132–827) with an extremely significant E value of 6.4 e^-97 ^[40]. BLAST analysis of the derived amino acid sequence of the *S. schenckii *SSPLA_2_, showed a phospholipase domain extending from amino acids 177 to 750 [39]. Pfam analysis shows similar results, and in this domain the PLA_2 _signature GXSG [G, S] (Pfam: Family PLA2_B PF 01735) is present as GVSGS in the active site (highlighted green in Figure 4B) [41,42]. The amino acids needed for catalytic activity R235, S263 and D553 are given in red in this same figure [43]. S263 is essential for the formation of arachidonyl serine needed for the transfer of the arachidonyl group to glycerol or to water. The amino acids D511 to L523, D583 to G595 and D738 to A750 (highlighted in yellow) comprise putative EF hand domains of the protein (76% identity, probability, 3.33e^-06^). In Figure 4B a putative calmodulin binding domain was identified from amino acids Q806 to L823 using the Calmodulin Target Database [44] and highlighted in gray. A serine protease, subtilase family, aspartic acid active site motif was identified using Scan Prosite with an E value of 5.283e-^07 ^from amino acids 549 to 559 and is shaded in blue green in Figure 4B[45]. This motif is characteristic of both yeast and fungal cPLA_2 _homologues [43].

**Figure 5:**
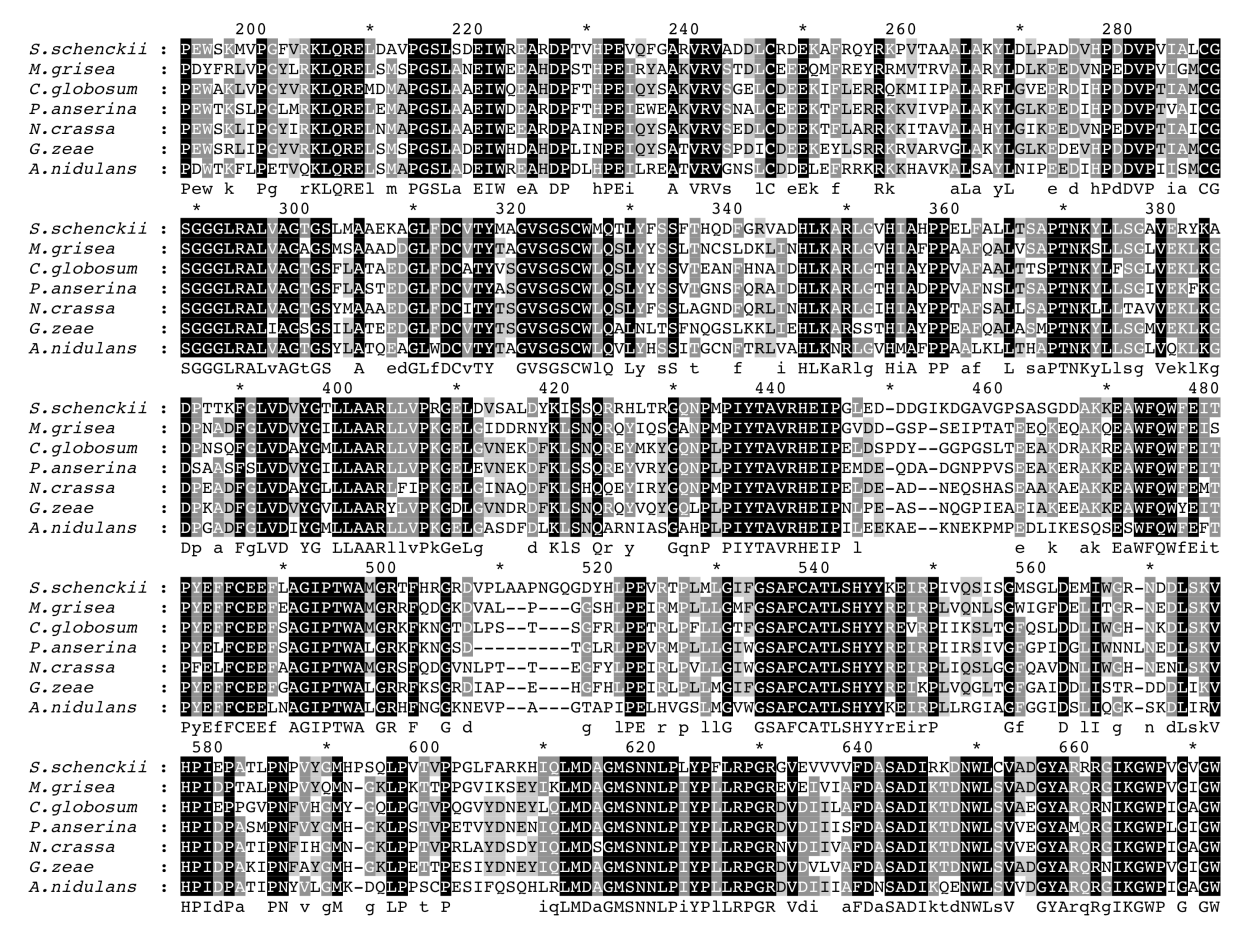
**Amino acid sequence alignments of SSPLA_2 _with other PLA_2 _homologues**. The *S. schenckii *SSPLA_2 _was aligned to other PLA_2 _fungal homologues as described in Methods. The fungal PLA_2 _used for the alignment were: *E. nidulans *(PLA_2_), *M. grisea *(hypothetical protein), *N. crassa *(PLA_2_), *C. globosum *(hypothetical protein), *P. anserina *(hypothetical protein) and *G. zeae *(PLA_2_). The alignment was done using MCOFFEE and visualized using the program GeneDoc. Only the catalytic core of these proteins is shown in this alignment, from amino acids 192 to 611 (in reference to the multiple alignment position). The black shading with white letters indicates 100% identity, gray shading with white letters indicates 75–99% identity, gray shading with black letters indicates 50–74% identity.

Figure 5 shows the multiple sequence alignment of the derived amino acid sequence of *S. schenckii *PLA_2 _homologue to that of other PLA homologues or hypothetical proteins from *N. crassa*, *A. nidulans*, *M. grisea*, *Chaetomium globosum*, *Podospora anserina *and *Gibberella zeae*. This figure shows that the important domains are very similar, although variations occur in the N terminal and C terminal regions. The alignment shown includes only the catalytic domain, the complete alignment is given as additional material (Additional file 1).

### Effects of PLA_2 _effectors on the yeast to mycelium transition and the yeast cell cycle

**Figure 6:**
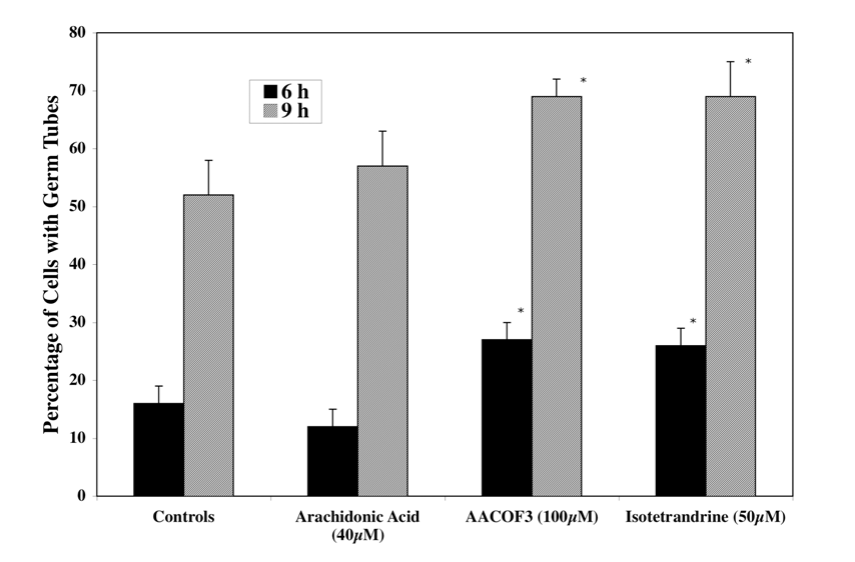
**Effects of SSPLA_2 _effectors on the yeast to mycelium transition**. Yeast cells grown, harvested, synchronized and selected by filtration as described in Methods were induced to form germ tubes in a basal medium with glucose at pH 4.0 and incubated at 25°C in the presence and absence of arachidonic acid (40 μM), AACOCF3 (100 μM; Nonadeca-4,7,10,13-tetraenyl-trifluoro-methyl ketone)) and isotetrandrine (50 μM; 6,6',7,12-tetra methoxy-2,2'-dimethyl-berbaman). All values are given as the average percentage ± one SD of at least three independent experiments. The Student's t test was used to determine the statistical significance of the data at a 95% confidence level. Values that differ significantly from those of the control at 95% confidence level are marked with an asterisk.

*S. schenckii *is not a genetically manageable organism, therefore, effectors of PLA_2 _were tested for their effects on the yeast to mycelium transition and the yeast cell cycle. Arachidonic acid is the primary product of cPLA_2 _activity on phospholipids, while AACOCF_3 _and isotetrandrine are inhibitors of PLA_2 _activity. AACOCF3 is a known competitive inhibitor of PLA_2 _[46]. It is an analogue of arachidonic acid and presumably binds directly to the active site of the enzyme. It is a potent and selective inhibitor of cytosolic phospholipase A [46]. Isotetrandrine on the other hand is an alkaloid that has been reported to interfere with G protein activation of PLA_2 _[47]. Figure 6 shows the percentage of yeast cells forming germ tubes in the presence and absence of arachidonic acid, AACOCF_3 _and isotetrandrine. This figure shows that these latter compounds significantly stimulated the yeast to mycelium transition at 6 and 9 h of incubation when the control cells are in the process of DNA synthesis and germ tube emergence [2]. The percent stimulation was approximately 68% and 33% at 6 h and 9 h of incubation in the presence of both AACOCF_3 _and isotetrandrine. In the presence of arachidonic acid a slight (25%) non-significant inhibition was observed at 6 h of incubation. The degree of stimulation caused by the addition of AACOCF_3 _and isotetrandrine was similar even though the mechanism of action of these compounds is completely different.

**Figure 7:**
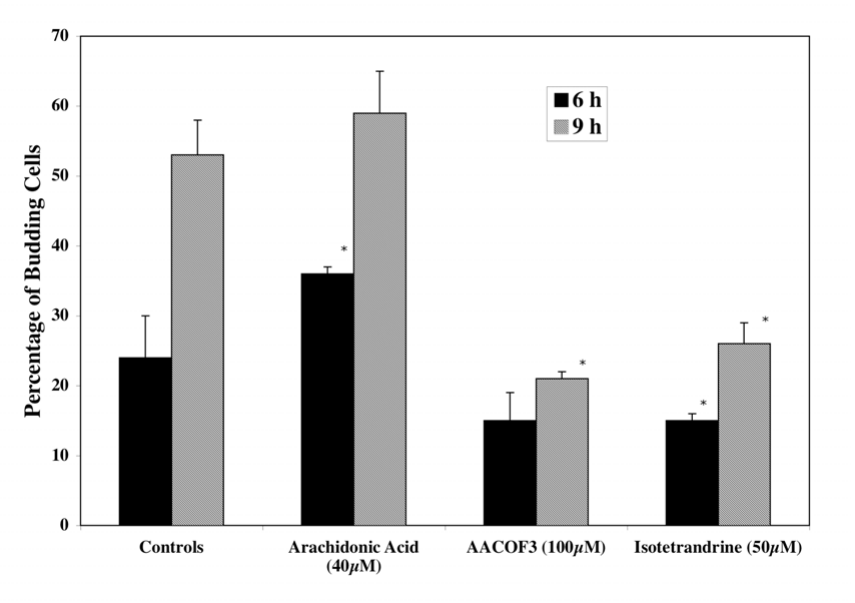
**Effects of SSPLA_2 _effectors on the yeast budding cycle**. Yeast cells grown, harvested, synchronized and selected by filtration as described in Methods were induced to re-enter the budding cycle in a basal medium with glucose at pH 7.2 and incubated at 25°C in the presence and absence of arachidonic acid (40 μM), AACOCF3 (100 μM; Nonadeca-4,7,10,13-tetraenyl-trifluoro-methyl ketone) and isotetrandrine (50 μM; 6,6',7,12-tetra methoxy-2,2'-dimethyl-berbaman). All values are given as the average percentage ± one SD of at least three independent experiments. The Student's t test was used to determine the statistical significance of the data at a 95% confidence level. Values that differ significantly from those of the control at 95% confidence level are marked with an asterisk.

Figure 7 shows the percentage of budding in yeast cells induced to re-enter the cell cycle in the presence and absence of arachidonic acid, AACOCF_3 _and isotetrandrine. The percent inhibition observed in the presence of both AACOCF_3 _and isotetrandrine was approximately 60% and 40% at 9 h of incubation, respectively. Arachidonic acid on the other hand significantly stimulated budding at 6 h of incubation (percent stimulation was 50%). At this time interval, control cells are initiating DNA synthesis [3].

## Discussion

The heterotrimeric G protein family ranks among the most important protein families identified as intracellular recipients of external signalling. The present study was conducted in order to describe new Gα subunit encoding genes in *S. schenckii*, identify any important protein interacting with this G alpha subunit and determine the effects on dimorphism in *S. schenckii *of the protein or proteins identified.

The results presented here, together with our previous report [19] corroborate the existence of more than one heterotrimeric G protein α subunit gene in *S. schenckii*. Unpublished results indicate that this protein is one of at least 3 Gα subunits present in *S. schenckii*. In this sense, *S. schenckii *is behaving more like the filamentous fungi and plant pathogens such as *N. crassa *[14], *C. parasitica *[48] and *M. grisea *[18], where genes that encode 3 different Gα subunits similar to the Gα class of animals rather than to the GPA group present in yeasts and plants. Computational sequence and phylogenetic analysis of the Gα subunits in filamentous fungi shows the existence of 3 distinct subfamilies of G protein alpha subunits [19]. According to the classification offered by Li and collaborators, SSG-2 belongs to Group III of the fungal G protein alpha subunits [49]. The Group III considered by them to be Gαs analogues because they positively influence cAMP levels although they have more sequence similarity to Gαi [49].

The nucleotide and amino acid sequence analysis of this new G protein α subunit gene are different from the previously identified *ssg-1 *gene. The nucleotide conservation of the coding region of *ssg-2 *is less than 50% when compared to that of the previously reported *ssg-1 *gene, confirming that *ssg-1 *and *ssg-2 *are two different genes (data not shown). The derived amino acid sequence of *ssg-2 *is 50% identical to that of SSG-1, but they have differences in the motifs that are characteristic of the G protein alpha subunits, the most important difference being that SSG-2 lacks the cysteine residue in domain 5 that characterizes the pertussis binding domain of SSG-1 (TCADT). For this reason, SSG-2 belongs to the Gα class but cannot be strictly considered a Gαi, even though it is 46% identical to mammalian Gαi class members. This shows the high degree of conservation in Gα subunits even among phylogenetically distant organisms.

The work done in order to identify the role of Gα subunits in the filamentous fungi has been mainly concerned with the phenotypes observed when these genes are knocked-out (as reviewed by [6]). In this paper a different approach was used. We wanted to identify important protein-protein interactions between SSG-2 and the complex signalling system that regulates the flow of information from the environment through the heterotrimeric G proteins into the cell in *S. schenckii*. Using the yeast two-hybrid technique we identified a cPLA_2 _homologue as interacting with SSG-2 in two independent experiments, using two different cDNA libraries. This SSG-2-PLA_2 _interaction was also confirmed by co-immunoprecipitation. Up to date, protein-protein interactions of these Gα subunits have not been reported in the pathogenic fungi, and the exact proteins with which these Gα subunits interact have not been identified. This is the first report of a cytosolic PLA_2 _homologue interacting with a G protein α subunit in a pathogenic dimorphic fungus, suggesting a functional relationship between these two important proteins. Other proteins interact with SSG-2 (unpublished results), but the SSG-2-PLA_2 _interaction is very important as it connects this G protein α subunit with both pathogenicity and lipid signal transduction in fungi [50].

This PLA_2 _homologue belongs to the Group IV PLA_2 _family that has been highly conserved throughout evolution. BLAST searches of the amino acid sequence of SSPLA_2 _against the *Homo sapiens *database shows that it is phylogenetically related to the human Group IVA PLA_2 _family. This same analysis using the fungal databases revealed that SSPLA_2 _is more closely related to the phospholipases of the filamentous fungi than to PLAB of yeasts. The similarity to both human and fungal phospholipases is found primarily in the catalytic domain with a great deal of variation contained in the first and last 200 amino acids. In the catalytic domain we find an important difference between SSPLA_2 _and the human homologues. The former has one continuous catalytic domain, rather than the more typical cPLA_2 _structure where two homologous catalytic domains are present, interspaced with unique sequences [43].

SSPLA_2 _lacks the C2 motif found in cPLA_2 _of higher eukaryotes. This domain is involved in the translocation of the enzyme to the membrane in response to an increase in intracellular calcium concentration [34]. Nevertheless, SSPLA_2 _has 3 putative EF hand motifs suggesting that it could also be calcium modulated. EF hand motifs are also present in the PLA_2 _homologues of *M. grisea, G. zeae*, *N. crassa *and *A. nidulans *in different areas of these proteins. It is interesting to note that *A. nidulans *PLA_2 _has been reported to be responsive to calcium even though it also lacks a C2 domain [51].

Also contributing to the possible modulation by calcium of this protein is the presence of a putative calmodulin binding domain [44]. As in the case of the EF hand-motifs, analysis of the PLA_2 _homologues of *M. grisea*, *N. crassa*, *G. zeae *and in *A. nidulans *show the presence of possible calmodulin binding domains in different areas of the proteins [44]. In *S. schenckii *the putative calmodulin binding domain is at the C terminal end of the protein, while in *M. grisea*, *N. crassa *and *G. zeae *it is within the first 150 to 250 amino acids.

In addition to the identification of PLA_2 _as interacting with SSG-2, we inquired as to the effects of PLA_2 _in *S. schenckii *dimorphism. As mentioned previously, PLA_2 _hydrolyses the *sn*-2 position of phospholipids, resulting in the release of lysophospholipids and free fatty acids. The most commonly released fatty acid is arachidonic acid. We tested the effects of exogenously added arachidonic acid on the kinetics of germ tube formation or the yeast cell cycle in *S. schenckii*. Our results show that exogenously added arachidonic acid had no significant effect on the kinetics of the yeast to mycelium transition, but a significant stimulation (50%) in the percentage of budding in cells induced to re-enter the yeast cell cycle was observed at 6 h of incubation in the presence of this compound. The observed stimulation of the yeast cell cycle by arachidonic acid is consistent with the inhibitory effects on this same cycle observed in the presence of AACOCF3 and isotetrandrine in *S. schenckii*, inhibitors of PLA_2_. These inhibitors have different mechanisms of action as stated previously. AACOCF3 is a competitive inhibitor of PLA_2 _[46] and an analogue of arachidonic acid, while isotetrandrine interferes with G protein activation of PLA_2 _[47]. Both AACOCF3 and isotetrandrine increased significantly the percentage of cells with germ tubes at 6 and 9 h after inoculation and decreased budding in cells induced to re-enter the yeast cycle. The AACOCF3 results are consistent with our hypothesis that PLA_2 _activity is needed for the yeast cell cycle in *S. schenckii*, specifically at the start of DNA synthesis [3]. Furthermore, the isotetrandine results support the hypothesis that the interaction of SSG-2 with PLA_2 _is required for these processes to occur.

It is of interest to note that we recently reported similar results in the presence of calmodulin inhibitor W7 and inhibitors of calcium-calmodulin kinase in *S. schenckii *[52]. Inhibiting calmodulin or calmodulin-dependent kinase also inhibited the re-entry of yeast cells into the cell cycle. We can speculate that by inhibiting the calmodulin dependent kinase we are also inhibiting the migration of cPLA_2 _to the membrane and/or its activation.

We cannot fully ascertain the functional consequences of the observed interaction between PLA_2 _and SSG-2 at this time. Future work will help us clarify this relationship. Nevertheless, two important processes that have a bearing in cell cycle progression have been identified as subjected to cPLA_2 _activity in other systems: 1) the production of biologically active molecules and 2) membrane remodeling [53].

There is very little information regarding the effects of the primary metabolites released from the action of PLA_2 _(arachidonic acid and lysophospholipids) in fungi, Arachidonic acid was reported to stimulate adenylate cyclase [54] in *S. cerevisiae*. If this is also true for *S. schenckii*, addition of arachidonic acid to the medium would be expected to stimulate the yeast cell cycle and this was what we observed. We had previously reported that dibutyryl derivatives of cAMP inhibit the yeast to mycelium transition in *S. schenckii *[4].

On the other hand, membrane remodeling is also an important function of enzymes such as phospholipases. This process is needed for cell cycle progression and fungal morphogenesis [53]. It has been reported in other systems that in order for the cell cycle to occur there must be a careful balance between membrane phospholipid synthesis and degradation. PLA_2 _has an important role in the maintenance of this balance [35,55]. The lipid composition of the membrane is also essential for the correct receptor-protein interactions and plays an important role in signal transduction. G proteins are usually in molar excess when compared to the GPRC's and a large number of inactive GDP-bound heterotrimeric G protein molecules must be available in receptor-rich domains associated to membrane lipids [56].

G proteins can also affect PLA_2 _activity by a number of different mechanisms such as: increasing the intracellular calcium concentration, transcriptional regulation and stimulation of phosphorylation through different protein kinases such as protein kinase C and MAP kinases (for a review see [57]).

The studies presented here constitute the first evidence of the interaction of G protein subunits of fungi with a phospholipase. These results establish for the first time a relationship between G proteins and the pathogenic determinants of fungi. The identification of such an important protein as partners of a G protein alpha subunit in fungi suggests a mechanism by which these G proteins can control pathogenicity in fungi. The existence of the interaction reported here may offer an explanation as to why fungi with decreased G protein alpha subunits such as *C. parasitica*, hypovirus infection [15] and *M. grisea *with disrupted Gα subunit gene, *magB*, exhibit reduced levels of virulence [18]. This information is essential if we are to understand the disease producing process of fungi. It will also help elucidate the signal transduction pathway leading from the G protein onward and will give us a better insight into signal transduction in pathogenesis and dimorphism in *S. schenckii*.

## Conclusion

We have shown the presence of a new G protein α subunit in *S. schenckii*, SSG-2. The cDNA sequence of the *ssg-2 *gene encoded a 355 amino acid Gα subunit of 40.90 kDa containing the 5 consensus domains present in all Gα subunits. The genomic sequence has four introns, whose positions are conserved in the other fungal homologues of this gene.

Yeast two-hybrid analysis using the complete amino acid sequence of SSG-2 identified a PLA_2 _homologue as an interacting partner of this G protein subunit. This 846 amino acid protein was encoded by an intronless gene. The 92.62 kDa protein encoded by this gene contained all the domains and amino acid residues that characterize cytosolic phospholipase A_2_.

PLA_2 _and other phospholipases in fungi have very diverse roles not only as virulence factors but also in membrane homeostasis and signal transduction. Inhibitor studies showed that this PLA_2 _homologue and its interaction with SSG-2 were necessary for the re-entry of *S*. *schenckii *yeast cells into the budding cycle suggesting a role for this important virulence factor in the control of dimorphism in this fungus and for the expression of the yeast form. The effects of PLA_2 _on the yeast cell cycle could be viewed as resulting from the generation of lipid messenger molecules or from membrane remodelling that affects the G1->S transition and G protein activity.

The relationship reported here between these two proteins, SSG-2 and SSPLA_2_, constitutes the first report of the interaction of a fungal phospholipase and a G protein subunit and the possible involvement of G protein in fungal virulence and morphogenesis.

## Methods

### Strains and culture conditions

*S. schenckii *(ATCC 58251) was used for all experiments. The yeast form of this fungus was obtained as described [2]. *S. cerevisiae *strains AH109 and Y187 were supplied with the MATCHMAKER Two-Hybrid System 3 (Clontech Laboratories Inc., Palo Alto, CA).

### Nucleic acids isolation

DNA and RNA were obtained from *S. schenckii *yeast cells as described previously using the methods of Sherman [58], and Chomczynski & Sacchi [59], respectively. Poly A^+ ^RNA was obtained from total RNA using the mRNA Purification Kit from Amersham Biosciences (Piscataway, NJ, USA).

### Sequencing the *ssg-2 *gene

#### Polymerase chain Reaction and Rapid amplification of cDNA ends (RACE)

*S. schenckii *DNA (100 ng) was used as template for polymerase chain reaction (PCR) with primers (100–200 ng) targeted to conserved motifs in Gα subunits. The primers used were: GESGKST (fw) 5' ggtgc(c/t)ggtga(a/g)tc(a/c)gg(a/t)aa(a/g)tc 3'; KWIHCF (rev) 5' aagcag tgaatccacttc 3'; TQATDT (rev) 5'gtatcggtagcttgggtc 3'; MGACMS (fw) 5' atggg ggcttgcatgagt 3' and KDSGIL (rev) 5' taggataccggaatctttg 3'. The Ready-to-Go™ PCR Beads (Amersham Biosciences) were used for PCR using the amplification parameters described previously [52].

PCR products were isolated and cloned using the TOPO TA Cloning System (Invitrogen Corp., Carlsbad, CA, USA) [19]. Plasmid preparations were obtained using the Fast Plasmid TM Mini technology from Eppendorf (Brinkmann Instruments, Inc. Westbury, NY, USA).

The 5' and 3' ends of the *S. schenckii *Gα subunit gene were obtained using SMART RACE (BD Biosciences, Clontech, Palo Alto CA, USA). All RACE reactions were carried out as described previously [19]. Primers for RACE were designed based on the sequence obtained previously. Nested primers were designed to improve the original amplification reactions. Bands from the 5' and 3' nested PCR, respectively, were excised from the gel, cloned and sequenced [19]. The following primers were used for 3' RACE: GSP2A (fw) 5' cttgaggaaagcagtcagaaccgaatgatg 3' and GSP2C (fw) 5' gtgaatcgggcacacctcaacttatatcct 3'. The following primers were used for 5' RACE: GSP1E (rev) 5' catcattcggttctgactgctttcctcaag 3'; GSP1D (rev) 5' aaagtcgcagtacgcacggatctcatcgct 3' and SSG-2 5 'UTR primer-1 (rev) 5' tagcagtagaatcttgcattctcgccgt 3' and SSG-2 5' UTR primer-2 (rev) 5' tcctcttcttctgctccacctcctcact 3'.

The complete coding sequence of the *ssg-2 *gene from cDNA and genomic DNA were obtained using reverse transcriptase polymerase chain reaction (RTPCR) and end to end PCR, respectively. The cDNA obtained using the RETROscript™ First Strand Synthesis kit (Ambion, Applied Biosystems, Foster City, CA, USA) was used as template. The following primers were used: MGACMS (fw)/KDSGIL (rev) primer pair. The sequence of these primers were the following: 5' atgggggcttgcatgagt 3' and 5' aggataccggaatctttg 3', respectively. For the genomic sequence PCR, DNA was used as template and the primers used were the same as those used for RTPCR. The PCR products containing the entire coding sequence, from both the cDNA and genomic templates were cloned and sequenced.

### Sequencing the *sspla*_*2 *_gene

#### Polymerase chain Reaction and Genome Walker

The 5' sequence of the PLA_2 _homologue was obtained using a combination of PCR and Genome Walker (Clontech Laboratories Inc., Palo Alto, CA, USA). Genomic DNA was used as template for PCR. For genome walking a Pvu II library of *S. schenckii *genomic DNA done as described by the manufacturer was used as template for the primary specific PCR reactions using the gene specific primers (GSP) and AP1 primer. The primary PCR reactions were used as template for nested PCR using nested gene specific primers (NGSP) and AP2 primer. The primers used were: YARRFA (NGSP, rev) 5' ccgagagacgatgcaaagcgacgggcgta 3'; SLLVFS (GSP and NGSP, rev) 5' agagaagacgaggagact 3'; GSLSDEIWRE (rev) 5' ctcgcgccaaatctcgtcggacagggatcc 3'; VHPEVQ (GSP, rev) 5' gaactggacttcggggtg 3'; LAKYLDLPA (NGSP, rev) 5' ggcagggaggtcgaggtacttggcgag 3'; (fw) 5' ctcgccaagtacctcgacctccctgccg 3'; DDVPVIA (rev) 5' aacgcaatcacgggcacgtcgtcgg 3'; GVSGSGC (fw) 5' ggagtgagcggttcatgctgg 3'; LYFSSFT (rev) 5' taaacgacgaaaagtacag 3'; PVGVGWPPA (GSP, rev) 5' cggccggcggccagcccacacccactgg 3'; PVGVG (fw) 5' ccagtgggtgtgggctg 3'; DDKIEQ (fw) 5' gacgacattgaacaggccaaagccgac 3'; DKIEQ (rev) 5' ttcaatcttgtcgtccgg 3'; ERHKRERL (rev) 5' cagccgctccctcttgtgccgctcctc 3'; NOVGGR (NGSP, rev) 5' ctccgacttaattaaaat 3'; 3' UTR primer (GSP, rev) 5' atgctgtgtcgccctccgac 3'. The AP1 and AP2 primers supplied by the manufacturer. The touchdown and nested PCR parameters used were those described previously [60].

### DNA sequencing and analysis

All sequencing reactions for the *ssg-2 *gene were conducted using the ABI PRISM™ 377 automated DNA sequencer (Applied Biosystems) and the Thermo Sequenase II Dye terminator Cycle Sequencing Premix Kit (Amersham Biosciences) as described previously [19]. Sequencing of the *sspla*_*2 *_gene products was done commercially using the SeqWright sequencing service (Fisher Scientific, Houston, TX, USA)

### Bioinformatics Sequence Analysis

The theoretical molecular weights were calculated using the on-line ExPASy tool http://www.expasy.ch/tools/. On-line Prosite Scan (Proscan Search) search was used to identify potential motifs present in SSG-2 and SSPLA_2 _http://npsa-pbil.ibcp.fr/cgi-bin/npsa_automat.pl?page=npsa_prosite.html[45]. The protein classification was performed using the PANTHER Gene and Protein Classification System http://www.pantherdb.org[40] and on-line Blocks Analysis Server http://blocks.fhcrc.org/blocks/blocks_search.html[37]. The calmodulin-binding domain was identified using the on line Calmodulin Target Database http://calcium.uhnres.utoronto.ca/ctdb/ctdb/sequence.html[44]. On-line database searches and comparisons for SSG-2 were performed using the Integrated Protein Classification (iProClass) database [61] and its BLAST algorithm implementation with a cutoff of 10^-7, ^a low complexity filter and the Blosum 62 matrix. The iProClass/UniProt accession numbers of the sequences used for the multiple sequence alignment of G protein subunits were: *S. schenckii *(SSG-2), Q8TF91; *M. grisea *(MAGA), O13314; *C. parasitica *(CPG2), Q00581; *N. crassa *(GNA3) Q9HFW7; *R. necatrix *(WGA1/RGA1), Q9HFA3; *E. nidulans *(GANB), Q9UVK8, and *S. schenckii *(SSG-1), O74259.

On-line database searches and comparisons for SSPLA_2 _were performed with the BLAST algorithm http://www.ncbi.nlm.nih.gov/BLAST/ with a cutoff of 10^-7^, a low complexity filter and the BLOSUM 62 matrix [39]. The Pfam analysis was done on-line using the using the Wellcome Trust Sanger Institute server http://pfam.sanger.ac.uk/[42]. The GenBank accession numbers for the multiple sequence alignment of phospholipases were: *A. nidulans *(PLA_2_), XP_663815; *S. schenckii *(SSPLA_2_), ACJ04517.1; *M. grisea *(hypothetical protein), XP_363597; *N. crassa *(PLA_2_), XP_962511; *C. globosum *(hypothetical protein) XP_001223932; *P. anserina *(hypothetical protein) XP_001909265, and *G. zeae *(PLA_2_), XP_382145.

Multiple sequence alignments were built using MCOFFEE http://www.igs.cnrs-mrs.fr/Tcoffee/tcoffee_cgi/index.cgi[62]. The alignments were visualized using the program GeneDoc http://www.nrbsc.org/downloads/.

### Yeast two-hybrid

MATCHMAKER Two-Hybrid System 3 was used for the yeast two-hybrid assay (Clontech Laboratories Inc., Palo Alto, CA) using all 3 different reporter genes for the confirmation for truly interacting proteins. For the construction of the bait plasmid, *ssg-2 *cDNA was obtained from poly A^+ ^RNA, transcribed and amplified by RT-PCR using the Ready-to-Go TM Beads (Amersham Biosciences). The RT-PCR product was amplified using primers containing the gene sequence and an additional sequence containing restriction enzyme sites, Xma I and BamH I at the 5' and 3' ends, respectively. The primers used were: Xma I-MGACMS (fw) 5' ccccggggatgggggcttgcatgagt 3' and DSGIL-BamH I (rev) 5' cgcggatccgcgctaggataccggaatctt 3'. The *ssg-2 *gene PCR product was cloned in frame into the linearized bait plasmid, pGBKT7 (Clontech Laboratories Inc.) using Quick T4 DNA ligase kit (New England Biolabs Inc., Ipswich, MA, USA) and amplified in *E. coli *by transformation. Sequencing corroborated the sequence, correct orientation, and frame of the inserted gene. The bait containing plasmid was isolated using Fast Plasmid™ Mini technology (Brinkmann Instruments, Inc.) and used to transform competent *S. cerevisiae *yeast cells (Y187). Competent *S. cerevisiae *yeast cells were transformed using the YEASTMAKER™ Yeast Transformation System 2 from Clontech (BD Biosciences, Clontech Laboratories Inc.). Tests for autonomous gene activation and cell toxicity were carried out also as described by the manufacturer.

Double stranded cDNA was synthesized from *S. schenckii *yeast cells Poly A^+ ^RNA using SMART™ Technology Kit (Clontech Laboratories Inc.). The cDNA's were amplified using Long Distance PCR and size selected using the BD CHROMA-SPIN™+TE-400 columns (Clontech Laboratories Inc.).

*S. cerevisiae *yeast cells AH109 were made competent using the lithium-acetate (LiAc) method mentioned above and transformed with SMART ds cDNA (20 μl) previously amplified by LD-PCR and the linearized pGADT7-Rec (Sma I-linearized plasmid). Transformants were selected in SD/-Leu plates, harvested and used for mating with the bait containing *S. cerevisiae *strain Y187. Mating of *S. cerevisiae *yeast cells strains Y187 (Mat-α) and AH109 (Mat-a) was done according to the manufacturer's instructions. The expression of three reporter ADE2, HIS3 and MEL1 genes in the diploids was used as confirmation for true interacting proteins. Diploids expressing interacting proteins were selected in triple drop out medium (TDO), SD/-Ade/-Leu/-Trp. Colonies growing in TDO medium were tested for growth and α-galactosidase production in quadruple drop out medium (QDO), SD/-Ade/-His/-Leu/-Trp/X-α-gal. Re-plating of these positive colonies into QDO medium was done at least 3 times to verify that they maintain the correct phenotype. Colony PCR was also done to corroborate the presence of both plasmids in the diploid cells using the T7/3'BD sequencing primer pair for the pGBKT7/*ssg-2 *plasmid and the T7/3'AD primer pair for the pGADT7-Rec library plasmid. The PCR products obtained with the T7 Sequencing Primer/3'AD Sequencing Primer pair were cloned and sequenced as described above.

### Co-immunoprecipitation (Co-IP)

*S. cerevisiae *diploids obtained in the yeast two-hybrid assay were grown in 125 ml flasks containing 25 ml of QDO for 16 h, harvested by centrifugation and resuspended in 4 ml containing phosphate buffer saline (400 μl) with phosphatase inhibitor (400 μl), deacetylase inhibitor (40 μl) (Active Motif North America, Carlsbad, CA, USA) and protease inhibitors cocktail (40 μl) (EDTA-free, Thermo Scientific, Pierce Biotechnology, Rockford, IL, USA). The cells were frozen in a porcelain mortar in liquid nitrogen, glass beads added and the cells broken as described previously [63]. The cell extract was centrifuged and the supernatant used for Co-IP using the Immunoprecipitation Starter Pack (GE Healthcare, Bio-Sciences AB, Bjorkgatan, Sweden) as described by the manufacturer. Briefly, 500 μl of the cell extract (1–2 ug of protein/ml) were combined with 1–5 μl of the anti-cMyc antibody (Clontech, Corp.) and incubated at 4°C for 4 h, followed by the addition of protein G beads and incubated at 4°C overnight in a rotary shaker. The suspension was centrifuged and the supernatant discarded, 500 μl of the wash buffer added followed by re-centrifugation. This was repeated 4 times. The pellet was resuspended in Laemmeli buffer (20 μl) and heated for 5 min at 95°C, centrifuged and the supernatant used for 10% SDS PAGE at 110 V/1 h. Pre-stained molecular weight standards were electrophoresed in outside lanes of the gel (BioRad Corporation, Hercules, CA, USA).

### Western Blots

Western blots were done as described by us previously [63]. The electrophoretically separated proteins were transferred to nitrocellulose membranes using the BioRad Trans Blot System^R ^for 1 h at 20 volts. After transferring, the nitrocellulose strips were blocked with 3% gelatin in TTBS (20 mM Tris, 500 mM NaCl, 0.05% Tween-20, pH 7.5) at room temperature for 30–60 min. The strips were washed for 5–10 min with TTBS. The TTBS was removed and the strips incubated overnight in the antibody solution containing 20 μg of antibody, anti-cMyc or anti-HA (Clontech, Corp.) was added to each strip. Controls where the primary antibody was not added were included. The antigen-antibody reaction was detected using the Immun-Star™ AP chemiluminescent protein detection system from BioRad Corporation as described by the manufacturer.

### Induction of the yeast to mycelium transition

The yeast form of the fungus was obtained from conidia as described previously [2]. Briefly, yeast cell were grown for 5 days from conidia in 125 ml flasks containing 50 ml of medium M with aeration at 35°C. These cells were filtered through sterile Whatman #1 filters (GE Healthcare Life Sciences). This procedure increases the concentration of undbudded singlets to approximately 90%. The cells were collected by filtration using Millipore filters GSWP04700 (0.2 μm) (Millipore Corp. Billerica, MA, USA), washed using basal medium with glucose and used for inoculation to give a final concentration of 10^5 ^cells/ml. These cells were induced to form germ tubes in the presence and absence of effectors of PLA_2 _activity in a basal medium with glucose at pH 4.0 and 25°C. Parallel cultures were inoculated with unbudded yeast cells and at 6 and 9 h after inoculation the content of a flask was filtered for the determination of the percentage of cells with germ tubes for each of the substances tested. These same yeast cells were inoculated to give a final concentration of 10^7 ^cells/ml and induced to re-enter the yeast cell cycle as described previously in the presence and absence of effectors of PLA_2 _in a basal medium with glucose at pH 7.2 and 25°C with aeration. At 6 and 9 h after inoculation samples were taken and the percentage of budding cells was recorded.

The following substances were tested for their effects on the yeast to mycelium transition and the yeast cell cycle: arachidonic acid (40 μM; AACOCF3 (100 μM; Nonadeca-4,7,10,13-tetraenyl-trifluoro-methyl ketone) [46] and isotetrandrine (50 μM; 6,6',7,12-tetra methoxy-2,2'-dimethyl-berbaman) [47]. These substances were obtained from Calbiochem, EMD Biosciences Inc. (Darmstadt, Germany). The results are expressed as the average percentage of cells with germ tubes or buds at 6 and 9 h of incubation ± one standard deviation of at least three independent determinations. The Student t test was used to determine the statistical significance of the data. A 95% confidence level was used to determine statistical significance.

## Authors' contributions

SVB carried out all the molecular biology studies concerning gene cloning and identification of *ssg-2 *gene, constructed a yeast cDNA library and did the first yeast two-hybrid analysis. SVB also conducted the PLA_2 _inhibition studies. WGV and LPS repeated the yeast two-hybrid analysis with a new cDNA library, identified PLA_2 _as an interacting protein for the second time and confirmed the results with co-immunoprecipitation. RGM carried out the sequence alignments and domain characterization of SSG-2 and PLA_2_. NRV designed the study, drafted the manuscript, completed the sequenced the *sspla*_*2 *_gene, participated in sequence identification, alignments and domain characterization. All authors have read and approved the final manuscript.

## Supplementary Material

Additional file 1 **Complete multiple sequence alignment of *S. schenckii *SSPLA_2 _to selected cPLA_2 _fungal homologues**. The complete multiple sequence alignment of fungal cPLA_2 _homologues to SSPLA_2 _as described in the methods is presented here. Click here for file
