# Supplementary material for: Cytosolic phospholipase A2: a member of the signalling pathway of a new G protein α subunit in Sporothrix schenckii
Source: BMC Microbiol. 2009 May 19;9:100. doi: 10.1186/1471-2180-9-100 (PMC2694196; doi:10.1186/1471-2180-9-100)
Supplement: Additional file 1 — Complete multiple sequence alignment of S. schenckii SSPLA2 to selected cPLA2 fungal homologues. The complete multiple sequence alignment of fungal cPLA2 homologues to SSPLA2 as described in the methods is presented here. [file 1471-2180-9-100-S1.pdf]

```
                      *        20         *        40         *        60         *        80         *
S.schencki : MAAH-RLPI-YA-----------------------------------------------RRFASS-LGGPSKTSSAVRTTATTAASLLAIGSLLVF :  46
M.grisea   : MSVLLRAGA-RSITLTGGVFPTEC--TSLGRGQRLTARSWNGYVG------YA---IGHRAMVT----RSPAEKRRIRFRRSWSSIV-VGTGILTY :  79
C.globosum : MALH-RGNI-IR------LNRLRFPIHSQGQIGFITTRTRPQLVNNGSGLPFDRQPP-RRCLILS----PRRHRSKVRATKLISVTL--TGALLAL :  81
P.anserina : MIPP-RSVLHRCLTGITRRRPLFSVTTTTRSPRRAP----FN--------------GRHGFFTSSRQGAEQKGSSKVGITRTVGLST--TCALLAL :  75
N.crassa   : MARL-GQLT-FAISSTGRRSPLARSLTQSGALLRAPARTKPSLLSFQ--------QTRHGFFASG----DNKPKPKTRVPRSLSAVL--LGSLLVF :  80
G.zeae     : MRHY-RGSL-RSLHRIPSRFNN----H-TRLPRRR---LTPR---------------QHRQFWTSHTKRDPKEEEQQRSTLPV-AVI--TGGLLIW :  68
A.nidulans : MGAA-RAFC-IRFAPPAA---LIYVG--GR-VLWSIEAARNNS---------------QLFLLHS----DPKAAYSTTSTQC-------------- :  55
             M    r                                                          s      k     r t            ll

              100         *       120         *       140         *       160         *       180         *
S.schencki : SLAPRDQHMRLDAAPALERRRTTKETQPSWAQ-N-G--KDDDEAS-LLSAGSESDMQKDKEGSTISAVGTWFR----DVANVDWSANPDRITGFIL : 133
M.grisea   : TLYPWEDRGQARQLATDQNR------RRIHP--N-H--KDGSHEQ-TTKA---SQSGS-GAWFNFAGSFEGLSTGLSKVADVDW--GTDKVLDYII : 157
C.globosum : WLSPTDAL-QHTDSRAKLA------PGNPSDE--------------DGAT---SSEDQ-SAWAKFSRNFGGLSF----VPSTDG--LSDRVVDMIM : 146
P.anserina : WLYPHESLTQLTKPLGLVA------DERRMSPDGVEIKVIKSTEKGRQKE---KREEE-SVWAGIAKGLEGFPS----IPGPP-ENITDMVVDFIL : 156
N.crassa   : SLNPTADVVQTFDSTDKTA------KTDRSSDTDL------FAQELESHG---KIDDS-STWSKLVDGFEGFSA----MTSAEAGKLSNKLVDLVL : 156
G.zeae     : WLYPSDDFAQLSGKQARLR------NDKDSGQD-------KSRDKSQNSE---SDADQ-SAWINFSRRFEAFST----LNSLEFSSFPDKIVNSLL : 143
A.nidulans : ------ARGQVHHSVGEGRP------GR-SNTD-------R--VEGSFHS---SEPDS-SIWSSMMQRFEGVKQSVG---SPEWIEL-DQLKNYII : 121
              l p     q                                           s     s w      feg                 d     i

                  200         *       220         *       240         *       260         *       280
S.schencki : PEWSKMVPGFVRKLQRELDAVPGSLSDEIWREARDPTVHPEVQFGARVRVADDLCRDEKAFRQYRKPVTAAALAKYLDLPADDVHPDDVPVIALCG : 229
M.grisea   : PDYFRLVPGYLRKLQRELSMSPGSLANEIWEEAHDPSTHPEIRYAAKVRVSTDLCEEEQMFREYRRMVTRVALARYLDLKEEDVNPEDVPVIGMCG : 253
C.globosum : PEWAKLVPGYVRKLQREMDMAPGSLAAEIWQEAHDPFTHPEIQYSAKVRVSGELCDEEKIFLERRQKMIIPALARFLGVEERDIHPDDVPTIAMCG : 242
P.anserina : PEWTKSLPGLMRKLQRELEMAPGSLAAEIWDEARDPFTHPEIEWEAKVRVSNALCEEEKTFLERRKKVIVPALAKYLGLKEEDIHPDDVPTVAICG : 252
N.crassa   : PEWSKLIPGYIRKLQRELNMAPGSLAAEIWEEARDPAINPEIQYSAKVRVSEDLCDEEKTFLARRKKITAVALAHYLGIKEEDVNPEDVPTIAICG : 252
G.zeae     : PEWSRLIPGYVRKLQRELSMSPGSLADEIWHDAHDPLINPEIQYSATVRVSPDICDEEKEYLSRRKRVARVGLAKYLGLKEDEVHPDDVPTIAMCG : 239
A.nidulans : PDWTKFLPETVQKLQRELSMAPGSLADEIWREAHDPDLHPEILREATVRVGNSLCDDELEFRRKRKKHAVKALSAYLNIPEEDIHPDDVPIISMCG : 217
             Pew k  Pg  rKLQREl m PGSLa EIW eA DP  hPEi   A VRVs  lC eEk f   Rk     aLa yL   e d hPdDVP ia CG

              *       300         *       320         *       340         *       360         *       380
S.schencki : SGGGLRALVAGTGSLMAAEKAGLFDCVTYMAGVSGSCWMQTLYFSSFTHQDFGRVADHLKARLGVHIAHPPELFALLTSAPTNKYLLSGAVERYKA : 325
M.grisea   : SGGGLRALVAGAGSMSAAADDGLFDCVTYTAGVSGSCWLQSLYYSSLTNCSLDKLINHLKARLGVHIAFPPAAFQALVSAPTNKSLLSGLVEKLKG : 349
C.globosum : SGGGLRALVAGTGSFLATAEDGLFDCATYVSGVSGSCWLQSLYYSSVTEANFHNAIDHLKARLGTHIAYPPVAFAALTTSPTNKYLFSGLVEKLKG : 338
P.anserina : SGGGLRALVAGTGSFLASTEDGLFDCVTYASGVSGSCWLQSLYYSSVTGNSFQRAIDHLKARLGTHIADPPVAFNSLTSAPTNKYLLSGIVEKFKG : 348
N.crassa   : SGGGLRALVAGTGSYMAAAEDGLFDCITYTSGVSGSCWLQSLYFSSLAGNDFQRLINHLKARLGIHIAYPPTAFSALLSAPTNKLLLTAVVEKLKG : 348
G.zeae     : SGGGLRALIAGSGSILATEEDGLFDCVTYTSGVSGSCWLQALNLTSFNQGSLKKLIEHLKARSSTHIAYPPEAFQALASMPTNKYLLSGMVEKLKG : 335
A.nidulans : SGGGLRALVAGTGSYLATQEAGLWDCVTYTAGVSGSCWLQVLYHSSITGCNFTRLVAHLKNRLGVHMAFPPAALKLLTHAPTNKYLLSGLVQKLKG : 313
             SGGGLRALvAGtGS  A  edGLfDCvTY  GVSGSCWlQ Ly sS t   f   i HLKaRlg HiA PP af  L saPTNKyLlsg VeklKg
```

```
                   *     400         *     420         *     440         *     460         *     480
S.schencki : DPTTKFGLVDVYGTLLAARLLVPRGELDVSALDYKISSQRRHLTRGQNPMPIYTAVRHEIPGLED-DDGIKDGAVGPSASGDDAKKEAWFQWFEIT : 420
M.grisea   : DPNADFGLVDVYGILLAARLLVPKGELGIDDRNYKLSNQRQYIQSGANPMPIYTAVRHEIPGVDD-GSP-SEIPTATEEQKEQAKQEAWFQWFEIS : 443
C.globosum : DPNSQFGLVDAYGMLLAARLLVPKGELGVNEKDFKLSNQREYMKYGQNPLPIYTAVRHEIPELDSPDY--GGPGSLTEEAKDRAKREAWFQWFEIT : 432
P.anserina : DSAASFSLVDVYGILLAARLLVPKGELEVNEKDFKLSSQREYVRYGQNPLPIYTAVRHEIPEMDE-QDA-DGNPPVSEEAKERAKKEAWFQWFEIT : 442
N.crassa   : DPEADFGLVDAYGLLLAARLFIPKGELGINAQDFKLSHQQEYIRYGQNPMPIYTAVRHEIPELDE-AD--NEQSHASEAAKAEAKKEAWFQWFEMT : 441
G.zeae     : DPKADFGLVDVYGVLLAARYLVPKGDLGVNDRDFKLSNQRQYVQYGQLPLPIYTAVRHEIPNLPE-AS--NQGPIEAEIAKEEAKKEAWFQWYEIT : 428
A.nidulans : DPGADFGLVDIYGMLLAARLLVPKGELGASDFDLKLSNQARNIASGAHPLPIYTAVRHEIPILEEKAE--KNEKPMPEDLIKESQSESWFQWFEFT : 407
             Dp a FgLVD YG LLAARllvPkGeLg    d KlS Qr y   GqnP PIYTAVRHEIP l              e  k  ak EaWFQWfEit

                         *     500         *     520         *     540         *     560         *
S.schencki : PYEFFCEEFLAGIPTWAMGRTFHRGRDVPLAAPNGQGDYHLPEVRTPLMLGIFGSAFCATLSHYYKEIRPIVQSISGMSGLDEMIWGR-NDDLSKV : 515
M.grisea   : PYEFFCEEFEAGIPTWAMGRRFQDGKDVAL--P---GGSHLPEIRMPLLLGMFGSAFCATLSHYYREIRPLVQNLSGWIGFDELITGR-NEDLSKV : 533
C.globosum : PYEFFCEEFSAGIPTWAMGRKFKNGTDLPS--T---SGFRLPETRLPFLLGTFGSAFCATLSHYYREVRPIIKSLTGFQSLDDLIWGH-NKDLSKV : 522
P.anserina : PYELFCEEFSAGIPTWALGRKFKNGSD---------TGLRLPEVRMPLLLGIWGSAFCATLSHYYREIRPIIRSIVGFGPIDGLIWNNLNEDLSKV : 529
N.crassa   : PFELFCEEFAAGIPTWAMGRSFQDGVNLPT--T---EGFYLPEIRLPVLLGIWGSAFCATLSHYYREIRPLIQSLGGFQAVDNLIWGH-NENLSKV : 531
G.zeae     : PYEFFCEEFGAGIPTWALGRRFKSGRDIAP--E---HGFHLPEIRLPLLMGIFGSAFCATLSHYYREIKPLVQGLTGFGAIDDLISTR-DDDLIKV : 518
A.nidulans : PYEFFCEELNAGIPTWALGRHFNGGKNEVP--A---GTAPIPELHVGSLMGVWGSAFCATLSHYYKEIRPLLRGIAGFGGIDSLIQGK-SKDLIRV : 497
             PyEfFCEEf AGIPTWA GR F  G d          g  lPE r p llG  GSAFCATLSHYYrEirP      Gf   D lI g  n dLskV

               580         *     600         *     620         *     640         *     660         *
S.schencki : HPIEPATLPNPVYGMHPSQLPVTVPPGLFARKHIQLMDAGMSNNLPLYPFLRPGRGVEVVVVFDASADIRKDNWLCVADGYARRRGIKGWPVGVGW : 611
M.grisea   : HPIDPTALPNPVYQMN-GKLPKTTPPGVIKSEYIKLMDAGMSNNLPIYPLLRPGREVEIVIAFDASADIKTDNWLSVADGYARQRGIKGWPVGIGW : 628
C.globosum : HPIEPPGVPNFVHGMY-GQLPGTVPQGVYDNEYLQLMDAGMSNNLPIYPLLRPGRDVDIILAFDASADIKTDNWLSVAEGYARQRNIKGWPIGAGW : 617
P.anserina : HPIDPASMPNFVYGMH-GKLPSTVPETVYDNENIQLMDAGMSNNLPIYPLLRPGRDVDIIISFDASADIKTDNWLSVVEGYAMQRGIKGWPLGIGW : 624
N.crassa   : HPIDPATIPNFIHGMN-GKLPPTVPRLAYDSDYIQLMDSGMSNNLPIYPLLRPGRNVDIIVAFDASADIKTDNWLSVVEGYARQRGIKGWPIGAGW : 626
G.zeae     : HPIDPAKIPNFAYGMH-GKLPETTPESIYDNEYIQLMDAGMSNNLPIYPLLRPGRDVDVLVAFDASADIKTDNWLSVADGYARQRNIKGWPVGIGW : 613
A.nidulans : HPIDPATIPNYVLGMK-DQLPPSCPESIFQSQHLRLMDAGMSNNLPIYPLLRPGRDVDIIIAFDNSADIKQENWLSVVDGYARQRGIKGWPIGAGW : 592
             HPIdPa  PN v gM  g LP t P        iqLMDaGMSNNLPiYPlLRPGR Vdi  aFDaSADIktdNWLsV  GYArqRgIKGWP G GW

                     680         *     700         *     720         *     740         *     760
S.schencki : PPADDPPEKTAQQLKDAGASAEAT-PDDKIEQAKADQAAHQRQNET-GSTTTTTKKDKRMGGAIGKQGSREAENAKAAAKELGYCTVWVGSTAEKV : 705
M.grisea   : PKETDPTEDTVKQLEDARANSQAE-ADQKLQLAQHDQSQSQVEQPG-ENKDTAKGQDGQ----VKN-------KYERAAESLGYCTVWVGTTEERE : 711
C.globosum : PKS-SSPSVAASELEQAQAHSTAE-AEDKISQAKTDQAARHPHGPR-EPKPPGSQPMKP-----ESQ------RQTEAAHELGYCTVWVGTTQERS : 699
P.anserina : PKPEASAKETAKQLDKAESASSPSESQDRVADAKMEQAARQQQMQE------DDSSKKT-----EAE------KHQQASDELGYCTVWVGTTQERS : 703
N.crassa   : PKASASASTTARELKEAEEATEAE-ADNKLAQAKADQAIRHANDPKVKPEGDSDQSLMS-----QQE------KYDQTAHELGYCTVWVGTTEERR : 710
G.zeae     : PKPGEATSQVVEELDEAQAKSTRE-AESKLREAKKEQKELRQEAHE-EGKQVMAENDKT-----KFE------HGDQESGDLGYCTVWVGTNQERS : 696
A.nidulans : PRAADTLKETEQSLREPENISEE-ALNRRVSEAQ--QSSTHEHAHS-SSVTTKQTTTNP-----DFQ-------PSPADTDLDYCNVWVGTMQERL : 672
             Pk       t  L  a   s      k  Ak  Q                                          a   LgYCtVWVGtt Er
```

```
                *         780         *         800         *         820         *         840         *         860
S.schencki : STAQE-AAETEAAQSGDKRDKKGTKQVEMAPPIDESTQWRLTAPDAGLTLVYLPYLANEAKV------------P-----G-----------VDPA- : 772
M.grisea   : TEPPP-PLK----------------------TAEDTEKWRLMEPGAGIAVVYLPFVANDK-V------------E-----G-----------VDPA- : 755
C.globosum : TTEPP-PP-----------------------PVDDTSTWRLTHPDAGIAVVYLPFLANERAA------------P-----G-----------VDVA- : 743
P.anserina : SAPPP-PPK----------------------PIDDESSWRLMEPDAGIAVVYMPFLANEKKV------------P-----G-----------VDPA- : 748
N.crassa   : TDDPP-PP-----------------------PVDDTSSWKLMEPNAGIAVVYMPFLANEKVS------------S-----S-----------VDPA- : 754
G.zeae     : SKPPP-PSK----------------------ALSGDNSWQLMEPEAGIAVVYLPFISNDK-V------------P-----G-----------ISPG- : 740
A.nidulans : SDKEPPPSK---------RLFDPRQ--SSSDPDHRESDFHLMRPDAGIAVVYFPLLPNSSAPDLPSSTSLTKPSPSDQHSGSENNSSAQDTIDTTK : 757
                  pp p                      p      w Lm P AGiavVY PflaN                p     g           vdpa

                  *         880         *         900         *         920         *         940         *
S.schencki : -TS------------DFMSTWNFVYQPEEVDKAVALGAANFKEGADQIRATVRAVYERKKQMRLAAEERHKRERLRRLVKQ---------EGNHFN : 846
M.grisea   : -TS------------DYMSTWNFVYTPEQVDKVVSLAKANYGEGRDQIKATVRAVYERKKIMREKREEQTRREHYRRLVRLGIADKLG--EGDHFS : 836
C.globosum : -VS------------DYMSTWNFVYTPPQIDEVIALARANYSEGRAQIRATVRAVYERKKKRRLEKERRAERAELRRLVRRGDVSGLG--EGDHFS : 824
P.anserina : -GS------------EYMSTWNFVYSPEDVEGVVRLARANYEEGRGQIKATVRAVYERKKRRREEHARRVKEEAWRRVIRGGRAGKVGVEGGDQFS : 831
N.crassa   : -SS------------DYMSTWNFVYTPEQVDQVVALARANYDEGKEKIRATVRAVYERKKNSRLEREKRMAEERQRRKVRLGIDGKLG--EGDHFS : 835
G.zeae     : -TT------------DYLSTWNFIYTPEQIDNVVELARANYNEGKQQIRDTIRGVYERKKKLREEAEKAQREDRYRSLMRRGEGVRLG--EGDHFS : 821
A.nidulans : PLSPHPGTINPNVDDFLSTWNFVYTPEQIDAVVGLAKANFAQGEQQVKRVVRAVYERKKKDRLQREERMHGPHKTGLVL---------------S : 837
               s            dymSTWNFvYtPeq d vv La ANy eG  qi atvRaVYERKK  R   e r      rrlvr g     g  egdhfs
```
